# Horizontal Saccadic Velocity in Patients with Exotropia before and after Unilateral Resection and Recession Surgery

**DOI:** 10.1155/2019/1374917

**Published:** 2019-02-13

**Authors:** Miharu Mihara, Atsushi Hayashi, Kazuya Fujita, Ken Kakeue, Ryoi Tamura

**Affiliations:** ^1^Department of Ophthalmology, Graduate School of Medicine and Pharmaceutical Sciences, University of Toyama, 2630 Sugitani, Toyama 930-0194, Japan; ^2^Department of Integrative Neuroscience, Graduate School of Medicine and Pharmaceutical Sciences, University of Toyama, 2630 Sugitani, Toyama 930-0194, Japan

## Abstract

**Purpose:**

The effects of strabismus surgery on eye movement are not known in detail, as few studies have compared saccade velocities before and after strabismus surgery. In this study, horizontal saccades were recorded using an eye-tracker in patients with only exotropia to compare the peak velocities (PVs), before and after undergoing strabismus surgery of the same type (unilateral resection and recession).

**Methods:**

Horizontal saccades of monocular vision were recorded using an eye-tracking device in 18 patients with exotropia and 20 normal subjects. All patients were examined using the same method after strabismus surgery.

**Results:**

The PVs of adduction and abduction in the patients were higher than those in the normal subjects (in dominant eye, *P*=0.032 for adduction and *P*=0.049 for abduction; in nondominant eye, *P*=0.016 for adduction and *P*=0.037 for abduction). Following the surgery, the PVs of abduction of the surgical eye (nondominant eye) decreased to the level of the normal subjects (*P*=0.016). However, there were no correlations between changes in the PVs and the extent of surgery (resection and recession).

**Conclusion:**

Strabismus surgery normalized the patient's increased PV in the operated eye for abduction of horizontal saccade. Not only peripheral (extraocular muscle) but also central sensory-motor mechanisms may be involved in the changes in PV of horizontal saccades, both of which could result from the improvement of the primary eye position.

## 1. Introduction

Saccadic eye movements are important for rapid gaze shifting to visual targets. The peak velocity (PV) of saccades is an important parameter for saccade evaluation. The PV correlates with the amplitude of saccades, and the normal range of the PV has already been determined [1, 2]. The PV of horizontal saccades reportedly reaches adult values by the age of 4.5 [3]. A previous study has reported no difference in the mean velocity of horizontal saccades between patients with horizontal strabismus and normal subjects [4].

However, few studies have compared saccade velocities before and after strabismus surgery [4, 5]. Bucci et al. observed that the PV of horizontal saccades was significantly decreased after surgery for horizontal strabismus in some patients [5]. They suggested that strabismus surgery improved the velocity of convergence and of divergence [4]. However, these previous studies have tested the effects of correcting the eye position on conjugate eye movements in mixed subject populations, including different types of strabismus and some operative methods. In the present study, horizontal saccades were recorded noninvasively, using an eye tracking system, in patients with only exotropia (XT), before and after undergoing strabismus surgery of the same type (unilateral resection and recession), as well as in normal subjects. To differentiate eye movement directions, the PV of horizontal saccades for adduction and abduction was calculated separately in each subject. The data were compared between the 2 groups (normal subjects vs. presurgical patients) and pairwise compared between the patients pre- and postsurgery.

## 2. Materials and Methods

The present study was approved by the Institutional Review Board of the University of Toyama (approval # 27-159) and conformed to all local laws and to the principles of the Declaration of Helsinki. Written informed consent was obtained from each participant after the experimental procedure was fully explained.

### 2.1. Participants

Twenty normal healthy adults (mean age: 29.9 years, range: 8–81 years, 8 women) and 18 patients with XT (mean age: 26.8 years, range: 6–80 years, 8 women) volunteered to participate in the study. All participants without dyscoria could clearly observe the visual target 40 cm away, with either the naked eye or soft contact lenses (corrected visual acuity of 1.0 or greater). All patients underwent surgery (resection of medial rectus muscle and recession of lateral rectus muscle) on their nondominant eyes, performed by 2 surgeons (MM and KF). The deviation angles near (30 cm) and far (5 m) were measured using the alternate prism cover test. The dominant eye was determined using the hole-in-card test [6]. Patients with constant XT (acquired, concomitance, not consecutive) were considered eligible if exophoria (or orthophoria) and stereopsis, based on the Titmus stereo test, were achieved after the surgery. All patients were examined before and again 6–12 weeks after surgery.

### 2.2. Stimulus Presentation and Examination Paradigm

The target consisted of a red inner circle (0.2° in diameter) superimposed at the center of a black circle (1.5° in diameter) and a white cross, in accordance with the study by Thaler et al. [7]. A computer monitor (Diamondcrysta®, RDT222WM-S, Mitsubishi, Tokyo) displaying the target was located at 40 cm from the participant's eye. The monitor luminance was 190 cd/m^2^, the resolution was 1,680 × 1,050 pixels, and the refresh rate was 60 Hz.

The participants sat on a chair in front of a table with their heads stabilized in a head positioner. They were examined under the following two conditions: monocular viewing with the dominant eye and monocular viewing with the nondominant eye; the other, nonviewing eye covered by an infrared filter (IR-76®, Fujifilm, Tokyo, Japan). They were instructed to fixate their gaze on a target located in the center at the beginning of the test trial, to fixate on the new target as rapidly as possible when it appeared at either 15° rightward or 15° leftward randomly, and finally to return their gaze to the original, center location when the latter target disappeared. Each subject performed five trials in each direction (adduction and abduction), with exclusion of saccade trials that included blinks or incorrect responses. A limited number of trials were used, as patients were examined during consultation hours and some participants were young children.

### 2.3. Eye Movement Recording

The horizontal (*X*) and vertical (*Y*) positions of both eyes were recorded using the ViewPoint EyeTracker® system (Arrington Research, Scottsdale, AZ, USA) at a sampling rate of 220 Hz, in accordance with the study by Crossland et al. [8–11]. This system consisted of two infrared cameras mounted on the head positioner, which sent images of the eye to a computer via a USB cable. The eye tracker recorded the subject's eye positions using the dark-pupil technique. These data were stored on a hard disk for offline data analysis. Prior to fixation stability testing, a 9-point grid (3 × 3 matrix) calibration and a subsequent validation procedure were performed for each subject using software supplied by the eye-tracker manufacturer (ViewPoint EyeTracker Software User Guide).

### 2.4. Data Analysis and Statistics

In the present study, only primary saccades, and not corrective saccades, were analyzed. The maximum value of the velocity waveform obtained from the primary differential values of the eye position in a saccade was defined as the PV. The onset of a primary saccade was defined as the time when the eye velocity exceeded 5% of the saccadic PV; the offset was set to when the eye velocity dropped below 10°/s, as is the previously reported standard [3, 4]. Data of eye movements that were in the wrong direction or that were contaminated with blinks were discarded. For each participant, the PVs (mean of 5 trials) for adduction and abduction of each eye were calculated.

The Mann–Whitney *U* test was used to compare the PVs for each dominant and nondominant eye between normal subjects and patients with XT, presurgically. The Wilcoxon signed-rank nonparametric test for paired samples was used to compare the PVs between pre and postsurgical parameters in patients with XT. For patients with XT, Spearman's rank-correlation between changes in the PV in the operated (nondominant) eye and the amount of surgery (resection or recession) was calculated. The JMP® statistical software package (SAS Institute, Cary, NC, USA) was used for the above tests; the significance level was set at *P* < 0.05.

## 3. Results

The presurgical exodeviation angle (mean ± SD) near and far were 45.9 ± 17.2 prism diopter (PD) (range: 16 PD to 95 PD) and 40.3 ± 18.1 PD (range: 14 PD to 87 PD), respectively. The surgical doses for the lateral rectus muscle and medial rectus muscle were 5.9 ± 1.4 mm (range: 4–8 mm) and 5.9 ± 1 mm (range: 4–8 mm), respectively. The postsurgical deviation angle (mean ± SD) near and far were 7.6 ± 10 PD (range: −10 PD to 30 PD) and 4.1 ± 5.1 PD (range: −10 PD to 18 PD), respectively. All the patients presented orthophoria or exophoria after surgery. No patient presented disturbance of adduction or abduction postsurgically. All patients showed stereopsis on the Titmus stereo test (Table 1).

### 3.1. Comparison of the PVs between Normal Subjects and Patients with XT

**Figure 1:**
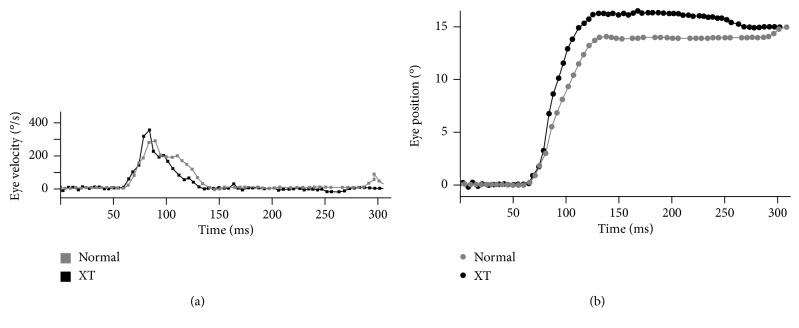
Typical example of an abducting saccade for right eye: eye velocity waveform (a) and primary differential value of eye position (b) for a normal subject (gray symbols) and a patients with exotropia (XT), before surgery (black symbols).

Figure 1 shows monocular recordings of typical sequences of horizontal saccades for a normal subject (gray symbols) and a patient with XT before surgery (black symbols). The shape of the primary saccade was smooth for most subjects in both groups, although some patients with XT sometimes exhibited overshoot Figure 1(b). The number of cases in which the saccades overshoot their targets was one for adduction and five for abduction in the normal group. In patients with XT before surgery, overshoot was noted in six cases for adduction and eight for abduction. The corrective saccade, which appeared 180 msec after the end of the primary saccade, facilitated an eye position that could reach the target (15° rightward).

**Figure 2:**
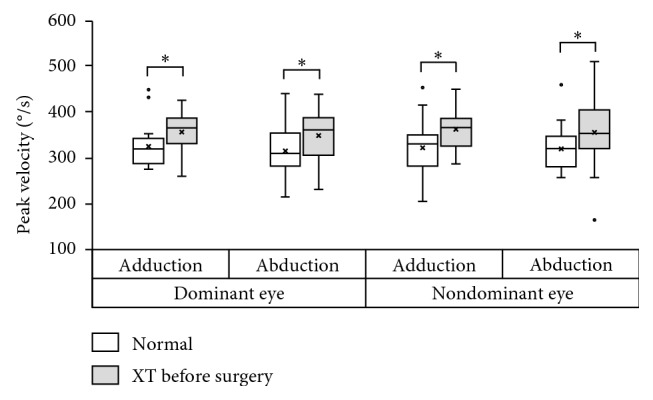
Comparison of horizontal saccadic velocity in normal subjects (white box) and presurgical patients with exotropia (XT, light gray box). In the dominant eye, the peak velocity (PV) was significantly higher in the XT group than in the normal group for both adduction and abduction. In the nondominant eye, the PV was similar to that of the dominant eye. The asterisk (^*∗*^) indicates *P* < 0.05.

The PV was significantly higher in the XT group than in the normal group, in either movement direction, for both eyes (Figure 2; in the dominant eye, *P*=0.032 for adduction and *P*=0.049 for abduction; in the nondominant eye, *P*=0.016 for adduction and *P*=0.037 for abduction).

### 3.2. Changes in Patients with XT after Surgery

#### 3.2.1. PVs in Adduction and Abduction

**Figure 3:**
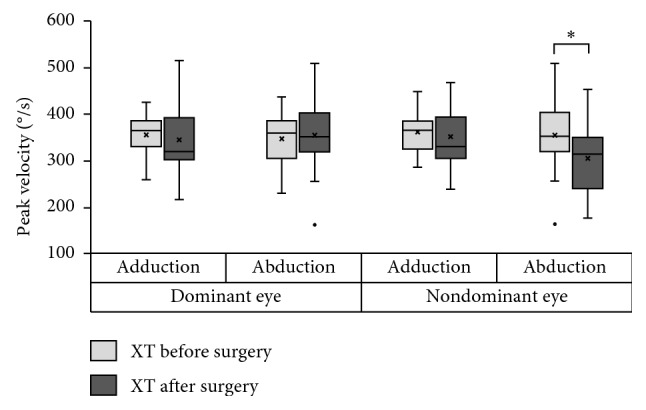
Comparison of presurgery (light gray box) and postsurgery (gray box) horizontal saccadic velocity in patients with exotropia. In the abduction of the nondominant eye (operated eye), the postsurgery peak velocity (PV) was significantly lower than the presurgery PV. The asterisk (^*∗*^) indicates *P* < 0.05.

For adduction, the PVs in the dominant eye tended to reduce after the surgery, although the difference did not reach statistical significance (*P*=0.55). No significant changes were observed in the nondominant eye (*P*=0.21) (Figure 3). For abduction, there were no significant changes in the PVs in the dominant eye after the surgery (*P*=0.39). The PVs in the nondominant eye clearly decreased after the surgery (*P*=0.016), such that the mean PV value approached that of normal subjects (Figure 3). There was no significant difference between the normal subjects and the patients with XT after surgery for abduction in the nondominant eye (*P*=0.55).

#### 3.2.2. Relationship between Changes in PVs and Extent of Surgery

**Figure 4:**
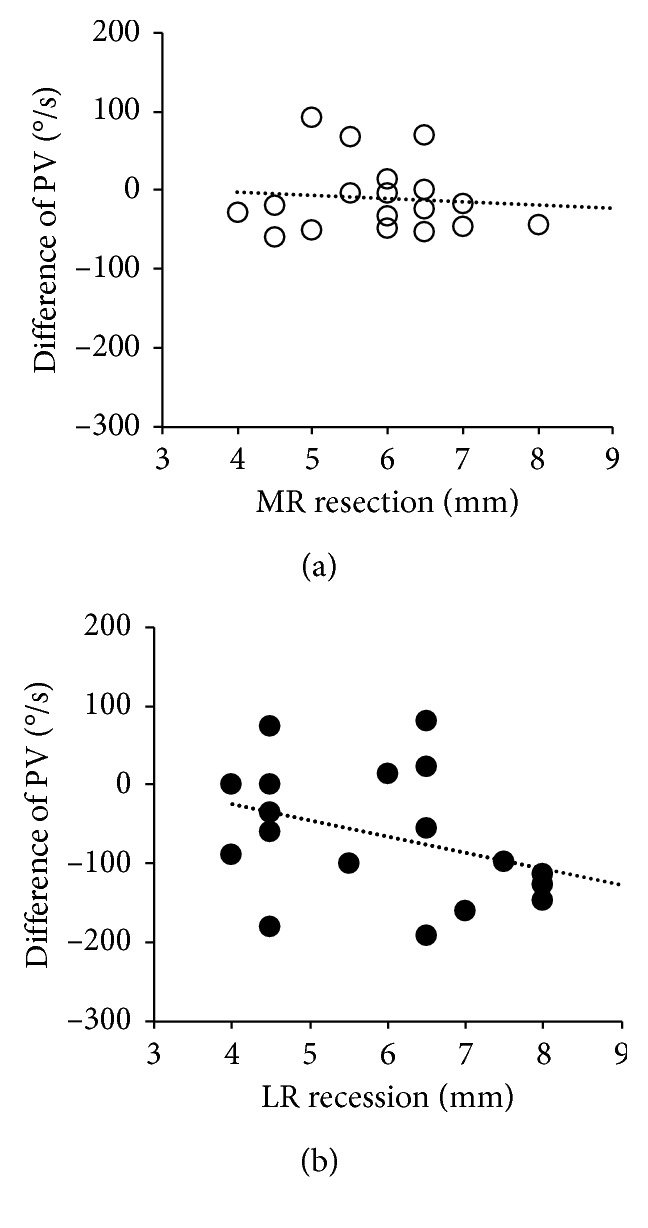
Relationships between changes in peak velocities (PVs) and extent of surgery. There was no correlation between changes in the PV and the extent of surgery in the operated, nondominant eyes (adduction and resection: white circle; abduction and recession: black circle). (a) Adduction; (b) abduction.

There was no correlation between changes in the PVs and the extent of the surgery in the operated, nondominant eyes (adduction: rs = −0.006, *P*=0.98; abduction: rs = −0.36, *P*=0.14) (Figure 4).

## 4. Discussion

We here investigated the effect of strabismus surgery on saccade velocities and showed that this surgery decreased these patients' increased PV in the operated eye for abduction of horizontal saccade.

The PV of horizontal saccades in normal subjects was almost the same as that previously reported [1, 2], while that in patients with XT was higher than that of the normal subjects, for both the dominant and nondominant eyes. It is presumed that the PVs were influenced by the accuracy of saccades (especially when overshoot saccades occurred), since the amplitude of saccades in patients with XT was slightly larger than in the normal subjects (data not shown). On the contrary, it is necessary to consider the cause due to the difference in the viewing condition. In a study by Niechwiej-Szwedo et al. [11], there was no significant difference in the PV of saccades between binocular viewing and monocular viewing in patients with horizontal strabismus without stereopsis, whereas in normal subjects and strabismus patients with stereopsis, the PV was lower during monocular viewing than during binocular viewing. Alternatively, as reported by Niechwiej-Szwedo et al. [11], the saccadic velocity in the normal subjects in the present study might have been decreased due to monocular viewing, because this was an unusual viewing condition for them. Conversely, the saccadic velocity of patients with XT, who were eligible for surgery, could not have been decreased because they had adapted to using monocular viewing over a long period of time. Difference of saccadic velocities between normal subjects and patients with XT could be caused by differences in both saccade accuracy and viewing condition.

Reduced tension of the extraocular muscles, which is the aim of strabismus surgery, may have resulted in changes of the PV. In the present study, however, there was no correlation between the changes in PVs after the surgery and the extent of resection/recession. In addition, the PV in adduction of the operated eye did not change after surgery. Kushner [12] performed a posterior fixation of the medial rectus in seven patients with convergence excess esotropia. They observed that postoperative saccadic velocity testing did not reveal the expected decrease in saccadic velocity, as the eye moved increasingly into the field of action of the operated muscle [12]. Surgical effects on saccadic velocity are likely to be different between the lateral rectus muscle and the medial rectus muscle. Bucci et al. [4] observed that the PV of horizontal saccades was significantly decreased after surgery for horizontal strabismus in three of eight patients. However, the directions of horizontal saccades were not reported in their result. Therefore, mechanisms other than extraocular (peripheral) factors are necessary to explain the change in the PV after surgery.

It has been reported that, in macaques with strabismus, the firing rate of neurons in the paramedian pontine reticular formation, the motor center for horizontal saccades, differed from that of normal monkeys [13]. Additionally, abnormal activities are observed in the abducens nucleus of macaques with strabismus [14]. Therefore, the patients in our study may have had some abnormal activities in these oculomotor centers before surgery. Orthophoria or exophoria achieved by strabismus surgery could have influenced and improved the central sensory-motor mechanisms within a few months after the surgery, since binocular viewing became easier as a result of the corrected primary eye position. This would have resulted in reduced PV in abduction. Although the present study does not provide direct evidence for this speculation, both peripheral and central sensory-motor mechanisms may be involved in the changes in the PV of horizontal saccades, both of which could result from the improvement of the primary eye position. On the other hand, Pullela et al. [15], in their study using two monkeys with strabismus, reported that the immediate changes in ocular alignment, saccades, and smooth pursuit after strabismus surgery gradually reverted to presurgical levels by six months after surgery. They concluded that immediate improvement in misalignment and changes in eye movement gains are likely due to contractility changes in the extraocular muscle, whereas longer-term effects are likely a combination of neural and muscle adaptation [15]. Therefore, longitudinal evaluation of saccades after strabismus surgery in humans will need to include investigation of neural responses after peripheral remodeling, since the data obtained a few months after strabismus surgery in the present study indicated that PVs change during the process of remodeling of extraocular muscles that have undergone resection and recession surgery.

## 5. Conclusions

The PVs of horizontal saccades in patients with exotropia were higher than those in the normal subjects. Following the surgery, the PVs of abduction of the surgical eye decreased to the level of the normal subjects; however, those of adduction were not changed. Also, there were no correlations between changes in the PVs and the extent of surgery. Therefore, not only peripheral (extraocular muscle) but also central sensory-motor mechanisms may be involved in the changes in PV of horizontal saccades, both of which could result from the improvement of the primary eye position. Longitudinal evaluation of saccades after strabismus surgery is necessary to investigate neural responses after peripheral remodeling in patients with strabismus.

## Figures and Tables

**Table 1:** Summary of patients with exotropia.

No.	Age	Sex	Dominant eye	Surgical dose (mm)	Deviation angle at near (PD)		Deviation angle at far (PD)		Stereopsis (second)	
					Before surgery	After surgery	Before surgery	After surgery	Before surgery	After surgery
1	27	M	LE	RE: MR4.0 LR4.0	30	16	14	4	40	40
2	31	M	LE	RE: MR6.5 LR6.5	57	0	72	0	100	60
3	35	F	LE	RE: MR7.0 LR7.5	40	6	50	8	400	100
4	21	M	LE	RE: MR5.5 LR4.5	45	12	40	2	RE sup	3000
5	70	F	LE	RE: MR6.0 LR5.5	58	0	42	−2	RE sup	100
6	6	M	LE	RE: MR6.0 LR6.0	45	16	35	10	50	50
7	22	F	LE	RE: MR6.0 LR7.0	59	−1	51	0	50	40
8	13	M	RE	LE: MR5.0 LR4.5	35	−10	25	−2	40	40
9	11	M	RE	LE: MR4.5 LR4.5	35	6	25	6	40	40
10	8	F	RE	LE: MR6.5 LR6.5	40	2	35	4	100	40
11	13	M	RE	LE: MR4.5 LR4.5	16	2	20	1	40	40
12	37	F	RE	LE: MR7.0 LR6.5	59	8	40	4	LE sup	3000
13	13	M	RE	LE: MR6.5 LR8.0	50	20	45	8	100	40
14	80	F	RE	LE: MR6.5 LR8.0	55	18	60	8	LE sup	200
15	53	M	RE	LE: MR8.0 LR8.0	95	30	87	18	50	50
16	20	F	RE	LE: MR5.5 LR4.0	52	20	30	10	40	40
17	10	M	LE	RE: MR6.0 LR6.5	30	−8	30	−10	40	40
18	12	F	RE	LE: MR5.0 LR4.5	25	0	25	4	140	50

M : male; F : female; RE : right eye; LE : left eye; MR: medial rectus; LR: lateral rectus; PD: prism diopter; sup: suppression.

## Data Availability

The data used to support the findings of this study are available from the corresponding author upon request.
